# Bacteremia Caused by a Putative Novel Species in the Genus *Erwinia*: A Case Report and Genomic Analysis

**DOI:** 10.3390/life15081227

**Published:** 2025-08-03

**Authors:** Jiwoo Lee, Taek Soo Kim, Hyunwoong Park, Jae Hyeon Park

**Affiliations:** 1 Department of Laboratory Medicine, Seoul National University Hospital, Seoul 03080, Republic of Korea; feste8545@snu.ac.kr (J.L.); kim.taeksoo@snu.ac.kr (T.S.K.); 2 Department of Laboratory Medicine, Seoul National University College of Medicine, Seoul 03080, Republic of Korea; bewithu2@snu.ac.kr; 3 Department of Laboratory Medicine, Seoul National University Boramae Medical Center, Seoul 07061, Republic of Korea

**Keywords:** *Erwinia*, bacteremia, catheter-associated infection, whole-genome sequencing, new taxa, clinical microbiology

## Abstract

We report a case of catheter-associated bloodstream infection caused by a putative novel species in the genus *Erwinia*, identified using whole-genome sequencing (WGS). A female adolescent receiving long-term home parenteral nutrition via a central venous catheter (CVC) presented with a fever. Gram-negative rods were isolated from two CVC-derived blood culture sets, while peripheral cultures remained negative. Conventional identification methods, including VITEK 2, Phoenix M50, MALDI-TOF MS, and 16S rRNA and *rpoB* gene sequencing, failed to achieve species-level identification. WGS was performed on the isolate using Illumina MiSeq. Genomic analysis revealed a genome size of 5.39 Mb with 56.8% GC content and high assembly completeness. The highest average nucleotide identity (ANI) was 90.3% with *Pantoea coffeiphila*, and ≤85% with known *Erwinia* species, suggesting that it represents a distinct taxon. Phylogenetic analyses placed the isolate within the *Erwinia* clade but separate from any known species. Antimicrobial susceptibility testing showed broad susceptibility. This case highlights the utility of WGS for the identification of rare or novel organisms not captured by conventional methods and expands the clinical spectrum of *Erwinia* species. While the criteria for species delineation were met, the phenotypic characterization remains insufficient to formally propose a new species.

## 1. Introduction

The genus *Erwinia* is Gram-negative, rod-shaped, aerobic, and motile bacteria that have been isolated from the environment. *Erwinia* spp. was first described by Winslow in 1920. *Erwinia* spp. originally belonged to the family *Enterobacteriaceae* and were reclassified into the family *Erwiniaceae* of the order *Enterobacterales* [1]. Within the genus *Erwinia*, 20 species have been validly published to date [2,3,4], with *Erwinia sorbitola* recently reported as a new species [5]. The genus *Erwinia* are usually plant-associated bacteria, and the most important species, *E. amylovora*, causes fire blight in the plant family *Rosacea* [6].

There are approximately 20 cases of human infection with *Erwinia* spp. to date, but most cases have been identified by biochemical methods [7]. *Erwinia herbicola*, which accounted for most of the human infection reports, was reclassified as *Pantoea agglomerans* along *Enterobacter agglomerans* [8]. Therefore, the only reports confirmed by molecular methods such as 16S rRNA sequence analysis are cervical lymphadenitis caused by *Erwinia tasmaniensis*-like organisms [9] and septic arthritis caused by *Erwinia billingiae* [7].

The identification of bacteria after culturing in clinical microbiological laboratories is the most fundamental task. Traditionally, biochemical assays have been used for this purpose. With the advancement of microbial taxonomy, however, the importance of a polyphasic taxonomic approach—which integrates phenotypic, genotypic, and phylogenetic data—has become increasingly recognized [10]. In clinical settings, matrix-assisted laser desorption ionization time-of-flight mass spectrometry (MALDI-TOF MS) is now widely used in conjunction with biochemical assays to enable rapid and accurate identification. 16S rRNA gene sequence analysis is initially used to identify bacteria using molecular methods, and alternative target sequencing such as *gyrB* and *rpoB* may be helpful according to the genus [11]. Next-generation sequencing (NGS) is becoming more and more widely used in clinical microbiology due to its high throughput, lower cost, and easier analysis. Therefore, identifying microorganisms by whole-genome sequencing (WGS) using NGS is the reference method [12,13]. In addition to species identification, WGS enables the analysis of antimicrobial resistance genes, virulence factors, strain typing, and plasmid content, as well as outbreak investigations [14,15]. Also, as more WGS data are accumulated and analyzed, the classification of bacteria is changing [1,16]. These genome-based approaches provide higher taxonomic resolution compared to traditional methods and underscore the growing importance of taking a polyphasic approach in bacterial classification [17].

Recent taxonomic frameworks emphasize the use of whole-genome-based metrics such as average nucleotide identity (ANI) and digital DNA–DNA hybridization (dDDH) for species delineation. According to the minimum standards proposed by Chun et al. and expanded by Riesco et al., a novel prokaryotic species is typically defined by an ANI value of less than 95–96% and a dDDH value below 70% compared with its closest relative [12,13]. These genome-based thresholds have largely replaced conventional DNA-DNA hybridization and are now widely accepted as the gold standard in prokaryotic systematics.

In parallel, only a limited number of human infections attributed to *Erwinia* species have been documented. However, many of these identifications were based solely on biochemical tests or partial 16S rRNA sequencing, which are often insufficient for accurate species-level discrimination. To date, only two published cases—cervical lymphadenitis by an *E. tasmaniensis*-like strain and septic arthritis by *E. billingiae*—have incorporated molecular analysis into species-level identification [7,9]. These highlight both the rarity of human infection by this genus and the importance of robust genomic tools for an accurate taxonomic assignment.

Here, we report a case of catheter-associated bacteremia caused by a Gram-negative rod that was identified as a putative novel *Erwinia* species based on whole-genome analysis. The phenotypic characteristics and antimicrobial susceptibilities of the strain were confirmed. WGS data were compared with the database and species were classified through phylogenetic tree analysis.

## 2. Materials and Methods

### 2.1. Microbial Culture and Identification

Blood culture bottles (BACTEC Peds Plus/F and Lytic/10 Anaerobic/F; Becton Dickinson, Sparks, MD, USA) were incubated in the BACTEC FX system. Positive blood cultures were subcultured onto blood agar and MacConkey agar plates and incubated at 35 °C for 16 h. Colonies were Gram-stained, and identification and antimicrobial susceptibility testing (AST) were performed using VITEK 2 (GN ID Card; bioMérieux, Marcy-l’Étoile, France). MALDI-TOF MS was conducted using the microflex LT system (Bruker Daltonics, Bremen, Germany), and the results were interpreted using the MALDI Biotyper library (version 6.0.0.0, 6903 MSPs), according to the manufacturer’s instructions. Identification scores were interpreted based on Bruker’s standard criteria, in which scores ≥2.0 indicate high-confidence species-level identification, scores between 1.7 and 1.99 indicate low-confidence identification, and scores <1.7 are considered unreliable.

For molecular identification, 16S rRNA gene amplification was performed using primers 27F/1492R for PCR and 785F/907R and 518F/800R for sequencing [11]. Additionally, *rpoB* gene sequencing was performed following the method described by Brady [18]. The resulting 16S rRNA and *rpoB* gene sequences were searched against the NCBI nucleotide database using BLAST 2.16.0 (https://blast.ncbi.nlm.nih.gov/, accessed on 17 June 2025). For 16S rRNA, the EzBioCloud database (CJ Bioscience, Suwon, Korea; version 2025.04.21) was also used [19]. The interpretation of sequence similarity followed the Clinical & Laboratory Standards Institute (CLSI) MM18-Ed2 guideline [11].

### 2.2. Biochemical Characterization

Biochemical profiling and enzyme activity were analyzed using GN ID Card by Vitek 2 (bioMérieux) and NMIC/ID-504 panel by BD Pheonix M50 (Becton Dickinson). The catalase and oxidase activities were tested. Triple Sugar Iron (TSI) agar was used to assess sugar fermentation and hydrogen sulfide production.

### 2.3. Whole-Genome Sequencing and Genomic Analysis

We implemented WGS using MiSeq (Illumina, San Diego, CA, USA) according to the protocol of the MAFGEN project of CJ Bioscience. The quality control of raw reads was conducted using FastQC v0.11.9 [20], and low-quality bases and adapter sequences were trimmed using Trimmomatic v0.39 [21]. The reads were then normalized to a depth of 100 using bbnorm v39.00 [22] and assembled with SPAdes v3.15.5 [23]. The assembly quality was evaluated by QUAST v5.2.0 and BUSO v5.8.2 with the *Enterobacterales* odb12 dataset [24,25]. The TrueBac™ ID-Genome system (CJ Bioscience) was used to identify the isolate and assess genomic similarity to type strains (https://www.truebacid.com/, Database version 20200206, accessed on 17 June 2025) [26]. In addition, k-mer-based taxonomic classification was conducted using KmerFinder v3.2 (https://cge.food.dtu.dk/services/KmerFinder/, accessed on 17 July 2025) with default parameters [27]. The assembled genome was queried against the CGE bacterial genome database, and the top hit and query coverage were recorded for comparison with the ANI-based results.

To evaluate genome-based taxonomic relatedness, ANI values were calculated using FastANI v1.34 with the minFraction parameter set to 0.1 [28]. Reference genomes belonging to the family *Erwiniaceae* were downloaded from the NCBI RefSeq database (https://www.ncbi.nlm.nih.gov/datasets/genome/, accessed on 17 July 2025). Only genomes from validly published species with correct names, as defined by the List of Prokaryotic names with Standing in Nomenclature (LPSN, https://lpsn.dsmz.de/, accessed on 17 July 2025), were included in the main analysis. Genomes with total assembled sizes below 1 Mb were excluded from the ANI matrix construction to ensure comparability and coverage consistency, as most Erwiniaceae genomes range from 3 to 5 Mb in size. The ANI values between the isolate and these reference genomes are presented in a heatmap and summary table. The heatmap was generated using the ComplexHeatmap package in R (v4.5.1) [29]. Additional comparisons with genomes of provisional or not validly published taxonomic status (e.g., Candidatus) are provided in Supplementary Table S2. Antimicrobial resistance genes were identified via the Comprehensive Antibiotic Resistance Database (CARD) (v4.0.1, https://card.mcmaster.ca/analyze/rgi, accessed on 17 June 2025) [30]. The genome was also screened for plasmid replicons and virulence genes using PlasmidFinder (v2.1, https://cge.food.dtu.dk/services/PlasmidFinder/, accessed on 17 June 2025) and VirulenceFinder (v2.0.3, https://cge.food.dtu.dk/services/VirulenceFinder/, accessed on 17 June 2025), respectively [31,32].

### 2.4. Phylogenetic and Taxonomic Analysis

Phylogenetic analyses were performed based on 16S rRNA and housekeeping gene (*rpoB* and *gyrB*) sequences, as well as WGS data. For gene-based phylogeny, the same set of reference genomes used in the ANI analysis was used to extract *rpoB* and *gyrB* sequences. The *rpoB* and *gyrB* gene sequences of *E. amylovora* were obtained from the NCBI reference genome and used as BLAST queries to identify homologous sequences. All *rpoB* sequences were successfully retrieved, while *gyrB* could not be extracted from *Erwinia mallotivora* due to a missing gene annotation in the NCBI genome assembly. For 16S rRNA analysis, sequences shorter than 1200 bp were excluded to ensure alignment quality and phylogenetic resolution. All sequences were aligned using MAFFT v7.525 with the --auto option and trimmed with trimAl v1.5.0 with the -automated1 setting [33,34]. Maximum-likelihood trees were constructed using RAxML-NG v1.2.2 with the GTR + G substitution model and 1000 bootstrap replicates [35]. For whole-genome-based phylogenetic analysis, genomes of type strains representing each genus in the family *Erwiniaceae*—including *Pantoea coffeiphila*—were analyzed using the Type Strain Genome Server (TYGS, https://tygs.dsmz.de/, accessed on 17 July 2025) with default parameters. The resulting trees were visualized using iTOL (Interactive Tree of Life) and the iTOL.toolkit [36,37].

### 2.5. Antimicrobial Susceptibility Testing

AST was performed using the AST-N224 panel (bioMérieux), NMIC/ID-504 panel (Becton Dickinson), Sensititre DKMGN panel (Thermo Fisher Scientific, Waltham, MA, USA), Etest (bioMérieux), and disk diffusion tests. The interpretations followed the CLSI M100 guidelines, applying breakpoints for *Enterobacterales* [38]. For Etest, a 0.5 McFarland bacterial suspension was inoculated onto Mueller–Hinton agar and incubated with Etest strips at 35 °C in 5% CO_2_ for 18 h. Disk diffusion tests were carried out under the same conditions. The colistin minimum inhibitory concentration (MIC) was determined using the Sensititre DKMGN panel, which employs broth microdilution methodology, interpreted according to the CLSI–EUCAST joint guidelines [39].

## 3. Results

### 3.1. Clinical Presentation

A female patient in her late teens was admitted through the emergency department with a one-day history of fever. She had previously undergone total colectomy and extensive small bowel resection due to pseudo-obstruction and had been on home total parenteral nutrition (TPN) for 10 years via a Hickman catheter. She had experienced multiple episodes of central line-associated bloodstream infections (CLABSIs), which were treated with antibiotics. Two weeks prior to this episode, she had been hospitalized for enterocolitis, treated with intravenous metronidazole, and discharged six days before the current admission.

At presentation, her vital signs were blood pressure 96/58 mmHg, pulse rate 67 bpm, respiratory rate 18 breaths/min, and body temperature 38.3 °C. Laboratory tests showed a white blood cell count of 8690/μL (absolute neutrophil count 7152/μL), hemoglobin 10.0 g/dL, platelet count 168,000/μL, and C-reactive protein 1.03 mg/dL. Two sets of blood cultures were obtained from both peripheral blood and the central venous catheter (CVC) before empirical cefotaxime was initiated.

After 14 and 23 h of culture bottle incubation, Gram-negative bacilli were isolated from the CVC samples. On hospital day 3, two repeat CVC blood cultures were negative, and the peripheral cultures obtained on hospital day 7 also yielded no growth. On hospital day 10, the CVC was removed and replaced. The catheter tip culture was negative. Hematochezia, likely due to small bowel obstruction, developed during hospitalization, prompting the re-initiation of metronidazole on hospital day 5. Cefotaxime and metronidazole were discontinued on hospital days 14 and 11, respectively, and the patient was discharged on day 19 following clinical improvement.

### 3.2. Conventional Phenotypic and Molecular Identification

Gram-negative rods were observed in the positive blood culture, and the isolate formed white and smooth colonies on the blood agar. The strain was catalase-positive and oxidase-negative, and it showed lactose fermentation on MacConkey agar. The Phoenix M50 system identified the microorganism as *P. agglomerans*, whereas the VITEK 2 GN ID card failed to provide a reliable identification. The isolate was subjected to MALDI-TOF MS analysis using the direct transfer method with the Bruker Biotyper system. The resulting identification scores were below the reliable threshold, with *Citrobacter braakii* (score 1.203) and *Lactobacillus kitasatonis* (score 1.196) being the first and second closest matches, respectively.

In 16S rRNA gene sequence analysis using the BLAST algorithm against the GenBank database (16S ribosomal RNA sequences), the isolate showed 98.50% identity (1448/1470 bp) with *E. billingiae* (RefSeq accession no: NR_104932.1), 98.30% (1444/1469 bp) identity with *E. tasmaniensis* (RefSeq accession no: NR_074869.1), and 98.62% identity (1428/1448 bp) with *P. coffeiphila* (RefSeq accession no: NR_178670.1). Despite the higher percent identity, *P. coffeiphila* was ranked third in the BLAST output, likely due to differences in alignment length and overall bit score, which influence the BLAST ranking algorithm. Analysis of the 16S rRNA gene sequence using the EzBioCloud database showed 99.02% identity (1427/1441 bp) with *P. coffeiphila* (accession no: KJ427829) and 98.97% identity (1443/1458 bp) with *E. billingiae* (accession no: JN175337). To further clarify the taxonomic position of the isolate, 16S rRNA gene sequence similarity was assessed using both the NCBI GenBank and EzBioCloud databases, and the results were interpreted according to the CLSI MM18-Ed2 guidelines [11]. As the 16S rRNA gene results were inconclusive for species-level identification, sequencing of the *rpoB* gene was performed as an additional marker for species-level identification. The *rpoB* gene sequence revealed 95.84% identity (991/1034 bp) with *Erwinia psidii* (GenBank accession no: CP132353.1) and 95.18% identity (987/1037 bp) with *Erwinia aphidicola* (GenBank accession no: CP188307.1). As accurate species-level identification could not be achieved using 16S rRNA and *rpoB* gene sequencing, WGS was performed to obtain a higher taxonomic resolution.

### 3.3. Whole-Genome Sequencing and Genome Quality

The assembled genome of the isolate was 5,392,830 bp in size, with a GC content of 56.78% and an average sequencing depth of 127.0×. The assembly consisted of 42 contigs, and the N50 value was 2,877,132 bp. The assessment of genome completeness using BUSCO (*Enterobacterales* odb12 dataset) showed 98.2% complete single-copy genes, 0.2% duplicated, 0.2% fragmented, and 1.5% missing, indicating high-quality genome assembly.

### 3.4. Whole-Genome Sequencing-Based Taxonomic Assignment

In the TrueBac^TM^ ID-Genome system (CJ Bioscience), the isolate *Erwinia* sp. strain SLM-02 was ambiguously identified as *Erwinia* sp., without assignment to a specific species. However, based on combined genomic similarity metrics including ANI, alignment coverage, and 16S rRNA identity, *Erwinia endophytica* and *E. aphidicola* were ranked as the closest matching species (Table 1). In addition, the k-mer-based analysis using KmerFinder identified *Erwinia persicina* as the closest match. However, the query coverage was only 3.9%, indicating distant similarity and supporting the need for high-resolution ANI and phylogenomic analyses (Supplementary Table S1).

Because TrueBacID indicated no close match (ANI < 85%), additional pairwise ANI analysis was performed using FastANI against a curated set of reference genomes representing the species in the family *Erwiniaceae* with correct names as defined by the LPSN. The highest ANI value was observed with *P. coffeiphila* (GCF_016909495.1), at 90.28%, while all of the other reference genomes showed ANI values below 83%, including *E. aphidicola* (82.75%), *E. rhapontici* (81.77%), and *E. persicina* (81.60%) (Table 2).

To ensure that no relevant genomes were overlooked, an extended ANI comparison including genomes with synonyms, provisional designations (e.g., Candidatus), and not validly published names was also performed. The top 20 matches from this expanded set are presented in Supplementary Table S2. No genome in this broader dataset exceeded the species-level ANI threshold or showed sufficiently high alignment coverage. These results fell well below the 95–96% threshold generally used for species delineation [12,13], supporting the classification of the isolate as a putative novel species distinct from all currently described members of the genus *Erwinia*.

**Figure 1:**
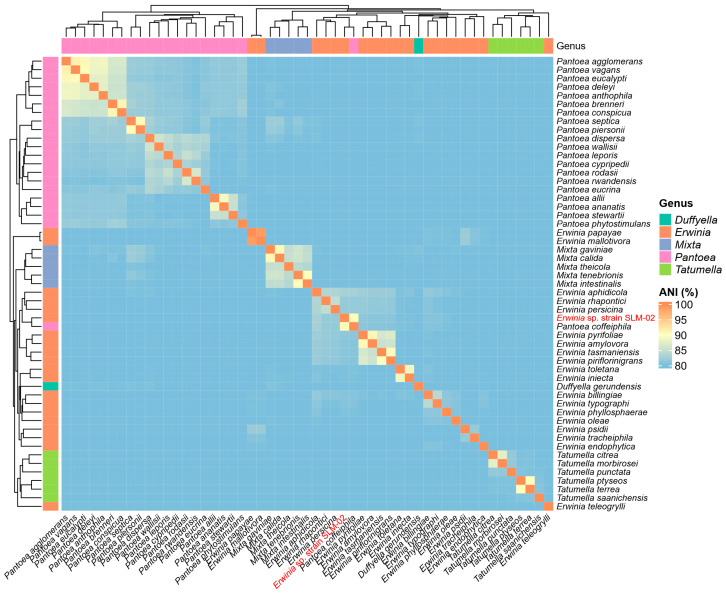
Pairwise ANI heatmap showing the isolate’s position within the *Erwinia* clade. Pairwise ANI values were calculated using FastANI (fragment length 3000 bp, minFraction 0.1) among representative genomes of *Erwinia* and related genera including *Pantoea*, *Mixta*, *Duffyella*, and *Tatumella*. Genomes with total assembled sizes below 1 Mb, such as those of *Buchnera* and *Phaseolibacter*, were excluded to ensure comparability, as most *Erwiniaceae* genomes range from 3 to 5 Mb in size. The isolate (*Erwinia* sp. strain SLM-02) is highlighted in red and clustered separately from all of the currently described *Erwinia* species.

Although the closest ANI match was a *Pantoea* species, pairwise ANI clustering (Figure 1) demonstrated that *P. coffeiphila* was positioned apart from other *Pantoea* genomes, suggesting possible misclassification. In contrast, the remaining *Erwinia* species formed a coherent clade, with the isolate located within this group but separated from all currently described species. This clustering pattern, together with the ANI results below the 95–96% species threshold, supports the interpretation that the isolate represents a putative novel *Erwinia* species.

Screening against the CARD detected no genes categorized as perfect hits and nine genes classified as strict hits, none of which conferred clinically relevant resistance. No known plasmid replicons or virulence genes were identified using PlasmidFinder and VirulenceFinder, respectively.

### 3.5. Phylogenetic Analysis

**Figure 2:**
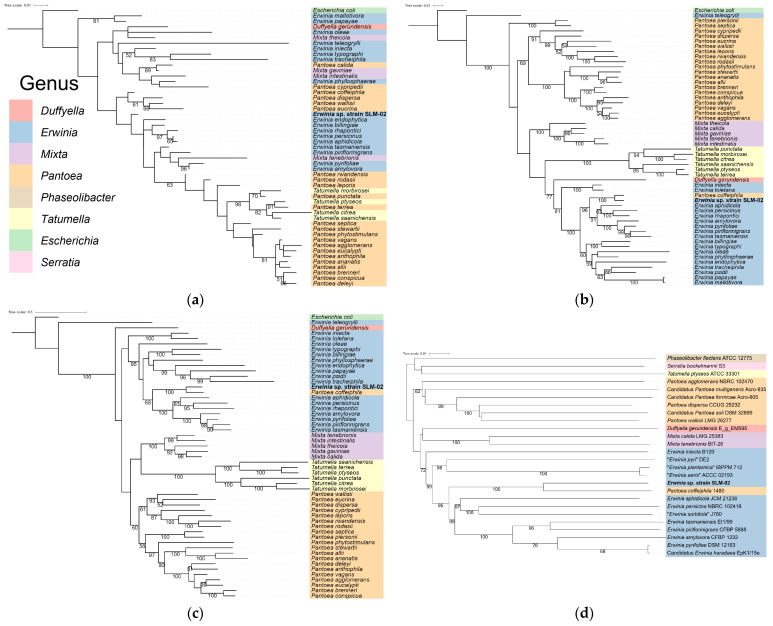
Phylogenetic analyses of the isolate within the family *Erwiniaceae*, based on 16S rRNA, *rpoB*, *gyrB*, and whole-genome sequences. (**a**) Maximum-likelihood tree based on 1343 aligned positions of 16S rRNA gene sequences from type strains of *Erwiniaceae*, including *Erwinia*, *Pantoea*, *Tatumella*, and *Mixta*. The tree was constructed using the GTR + G model, with *Escherichia soli* ATCC 11775^T^ (X80725) as the outgroup. (**b**) Maximum-likelihood tree based on 4027 aligned positions of *rpoB* gene sequences, constructed using the GTR + G model. (**c**) Maximum-likelihood tree based on 2405 aligned positions of *gyrB* gene sequences, constructed using the GTR + G model. (**d**) Whole-genome-based phylogenetic tree inferred using the Genome BLAST Distance Phylogeny (GBDP) method via TYGS, applying formula d5. Bootstrap support values (1000 replicates for (**a**–**c**); 100 replicates for (**d**)) are shown at each node. Only bootstrap values ≥50% are displayed. In all trees, the isolate characterized in this study is labeled as *Erwinia* sp. strain SLM-02.

To visualize evolutionary relationships beyond pairwise ANI, phylogenetic trees were reconstructed based on 16S rRNA, housekeeping genes (*rpoB* and *gyrB*), and whole-genome data. In the maximum-likelihood tree based on 16S rRNA sequences, the isolate clustered within the *Erwinia* genus, while *P. coffeiphila* grouped with other *Pantoea* species, indicating clear genus-level separation (Figure 2a). However, in the phylogenetic trees based on *rpoB* and *gyrB* sequences, *P. coffeiphila* was positioned within the *Erwinia* clade and showed close relatedness to the isolate, suggesting inconsistencies between the marker gene and 16S-based classifications (Figure 2b,c). Whole-genome-based phylogenetic analysis using the Type Strain Genome Server (TYGS) placed the isolate within the *Erwinia* genus, but as a distinct lineage separate from all of the currently described *Erwinia* species, including *E. billingiae*, *E. tasmaniensis*, and *E. aphidicola*, as well as from *P. coffeiphila*, which clustered nearby but outside the *Erwinia* genus.

### 3.6. Antimicrobial Susceptibility Profile of the Clinical Isolate

The AST results obtained by Etest, disk diffusion, and the Sensititre DKMGN panel are summarized in Table 3. The VITEK 2 AST-N224 panel and Phoenix M50 NMIC/ID-504 failed to produce valid results. The isolate was susceptible to most of the tested antimicrobial agents across all of the methods. Resistance was observed only to ampicillin. Ceftazidime–avibactam was initially reported as resistant in the NMIC/ID-504 panel, but repeat testing failed, and confirmatory testing using the DKMGN panel showed susceptibility.

## 4. Discussion

The genus *Erwinia* was originally classified within the family *Enterobacteriaceae*, but it was reclassified into *Erwiniaceae* following the reorganization of *Enterobacteriaceae* into the order *Enterobacterales*. *Erwiniaceae* has undergone several nomenclatural and taxonomic revisions. The *Erwinia herbicola–Enterobacter agglomerans* complex was reclassified as *Pantoea agglomerans* [8]. *Erwinia gerundensis* was described as a novel species of *Erwiniaceae* in 2016 [4] and was transferred to the newly established genus *Duffyella* in 2023, resulting in the updated name *Duffyella gerundensis* [40]. *Erwinia phyllosphaerae* was proposed as a novel species in 2022 [41]. Several new genera have also been proposed within *Erwiniaceae* over the past decade, including *Mixta* [42], *Kalamiella* [43], *Winslowiella* [44], and *Paramixta* [45]. Among these, only *Mixta* is currently recognized as a correct name according to the LPSN.

Human infections caused by *Erwiniaceae* members are rarely reported. The genera *Erwinia* and *Pantoea*, the principal members of this family, are primarily known as plant pathogens [46]. While human infections due to *E. herbicola* were historically described, the species has since been reclassified as *P. agglomerans*. Among *Pantoea* species, *P. agglomerans* is the most frequently implicated in human infections, with occasional cases attributed to *P. dispersa* and other species [47]. In contrast, human infections caused by *Erwinia* spp. are exceedingly rare, and only two cases have been identified using molecular methods such as 16S rRNA gene sequencing rather than biochemical testing [7,9]. Bonnet et al. analyzed only 521 bp of the 16S rRNA sequence in their study [7]. In closely related genera, identification based on *rpoB* or *gyrB* sequences is considered to be more accurate than 16S rRNA-based methods [18].

The VITEK 2 GN ID card can identify *P. agglomerans* and *Pantoea* spp., but its coverage of *Erwiniaceae* is limited. In contrast, the Bruker Biotyper library (version 6.0.0.0; 6903 MSPs) includes 35 spectra of 10 *Erwinia* species and 31 spectra of 7 *Pantoea* species. Nevertheless, the isolate could not be reliably identified at the genus level (score ≥1.7) using MALDI-TOF MS. Although one study reported the possibility of the species-level identification of *Erwinia* by MALDI-TOF MS [48], other study regarding the closeness of *Pantoea* with *Erwinia* claimed that 19% of *Pantoea* spp. identified by MALDI-TOF MS was classified as a different genus in *Enterobacterales* after *cpn60*-based molecular typing [49]. These findings suggest that MALDI-TOF MS may require further optimization and validation for the accurate identification of *Erwinia* spp., particularly through the inclusion of a broader range of reference spectra.

The application of WGS in clinical microbiology laboratories has gradually expanded. However, its routine use is still limited by a high cost, longer turnaround time, and the need for bioinformatic expertise. To address these limitations, several commercial platforms have emerged, including the TrueBac™ ID system. This WGS-based bacterial identification tool enabled species-level identification in 94% (34/36) of clinical isolates that could not be identified by MALDI-TOF MS, and four of those were predicted to be novel species [26]. Similarly, in our study, the isolate was determined to represent a putative novel *Erwinia* species based on its genomic features analyzed using the TrueBac™ ID platform.

The isolate showed approximately 99% 16S rRNA gene sequence identity with *P. coffeiphila* as well as several species within the genus *Erwinia*, indicating that 16S rRNA-based analysis alone was insufficient for species-level identification. Additional sequencing of the *rpoB* gene also failed to yield a definitive match, prompting WGS for higher taxonomic resolution. The ANI analysis revealed that the isolate shared the highest identity (90.3%) with *P. coffeiphila*, which is below the established species-level threshold of 95–96%. All other comparisons with *Erwinia* species yielded ANI values below 85%, further supporting its distinctiveness. However, both pairwise ANI clustering and whole-genome-based phylogenetic analysis consistently placed the isolate within the *Erwinia* clade. Interestingly, *P. coffeiphila* was also located within the same clade, rather than forming a cluster with other *Pantoea* species, raising the possibility that it may have been misclassified. Notably, *P. coffeiphila* was originally described without including *Erwinia* species in its phylogenetic framework [50], which may have contributed to its current taxonomic placement. Taken together, these findings support the classification of the isolate as a putative novel species within the genus *Erwinia* while also highlighting the need to re-evaluate the taxonomic status of *P. coffeiphila*.

CLABSI is a well-recognized and potentially serious complication in patients receiving long-term home TPN, particularly those with central venous catheters. According to data from the American Society for Parenteral and Enteral Nutrition’s registry and clinical guidelines, CLABSI occurs frequently in this population, especially among pediatric patients, and is associated with factors such as prolonged catheter use, anatomical alterations of the gastrointestinal tract, and underlying intestinal failure [51,52]. Common pathogens include coagulase-negative *Staphylococcus*, methicillin-susceptible *Staphylococcus aureus*, *Klebsiella* spp., and other Gram-negative bacilli, as widely reported in catheter-related bloodstream infections (CRBSIs) [53]. In this case, the patient presented with fever, and the same organism was isolated from two CVC blood cultures, fulfilling the criteria for CLABSI. However, the case did not meet the definition of CRBSI because differential time-to-positivity was not available and the catheter tip culture was negative. Although CLABSIs are thought to overestimate actual central-line bloodstream infections compared to CRBSIs because they are based on exclusion diagnosis, their importance remains significant due to similar mortality rates and hospital lengths of stay as CRBSIs [54,55]. In this case, the isolate was identified as a putative novel *Erwinia* species, and its role as a pathogen remains unclear. Given the patient’s long-term catheter use, extensive intestinal resection, and history of recurrent CLABSI, the organism is presumed to represent an opportunistic pathogen with low intrinsic virulence. This interpretation is further supported by the absence of known resistance or virulence genes in genomic analyses and by phenotypic susceptibility to most tested antimicrobials. Such organisms may cause clinically significant infections in immunocompromised patients or those with compromised mucosal barriers.

A limitation of this study is the absence of a detailed phenotypic characterization of the isolate. It has been noted by microbial taxonomists that taxonomy should not rely solely on genomic data due to concerns such as sequencing quality, lack of standardization, and limited applicability to disciplines that depend on phenotypic traits [56]. Accordingly, the polyphasic approach—which integrates morphological, physiological, and biochemical features—remains essential in modern microbial taxonomy, and we do not propose a formal species name in this study. Nevertheless, WGS provides high-resolution and quantitative information for bacterial identification, and our WGS-based approach contributes meaningful insights into the taxonomic and clinical interpretation of this rare isolate.

In summary, we report a case of CLABSI caused by a Gram-negative bacillus that was identified as a putative novel species within the genus *Erwinia* using WGS. The 16S-based identification yielded conflicting results depending on the database used, and the ANI analysis did not support species-level identity with any known taxa. Pairwise ANI clustering and whole-genome-based phylogenetic analysis consistently placed the isolate within the *Erwinia* clade, distinct from all of the described species. These findings support the classification of the isolate as a putative novel species within *Erwinia*. As WGS becomes increasingly accessible, genome-based identification is expected to play a critical role in the discovery and classification of rare and novel pathogens in clinical microbiology.

## Figures and Tables

**Table 1:** Top 10 matches from the TrueBac™ ID-Genome system based on whole-genome ANI and 16S rRNA similarity with the isolate.

No.	Closest Matching Species	Whole-Genome ANI (%)	ANI Alignment Coverage (%)	16S rRNA Identity (%)
1	*Erwinia endophytica*	84.95	37.2	98.45
2	*Erwinia aphidicola*	85.01	37.4	98.35
3	*Erwinia rhapontici*	84.53	30.5	98.35
4	*Erwinia persicina*	84.47	31.7	98.35
5	JFGT_s	84.11	18.1	97.11
6	RHUM_s	84.25	21.6	98.76
7	KZ478080_s	83.81	8.3	99.18
8	*Erwinia billingiae*	84.17	22.9	99.18
9	*Pantoea coffeiphila*	83.92	11.2	99.06
10	ALXE_s	83.81	9.3	98.76

Abbreviation: ANI, average nucleotide identity. Coverage indicates the percentage of aligned genome fragments in the query. JFGT_s and RHUM_s are provisional designations of unnamed strains included in the TrueBac™ database.

**Table 2:** Average nucleotide identity (ANI) and alignment coverage between the isolate and the top 10 reference genomes from the family *Erwiniaceae*, based on FastANI.

No.	Species Name	RefSeq Accession	ANI (%)	No. of Aligned Fragments	Total Query Fragments	ANI Alignment Coverage (%)
1	*Pantoea coffeiphila*	GCF_016909495.1	90.28	1566	1786	87.68
2	*Erwinia aphidicola*	GCF_037149315.1	82.75	994	1786	55.66
3	*Erwinia rhapontici*	GCF_020683125.1	81.77	964	1786	53.98
4	*Erwinia persicina*	GCF_019844095.1	81.60	959	1786	53.70
5	*Erwinia pyrifoliae*	GCF_002952315.1	81.06	691	1786	38.69
6	*Erwinia amylovora*	GCF_043228865.1	80.93	688	1786	38.52
7	*Erwinia piriflorinigrans*	GCF_001050515.1	80.86	686	1786	38.41
8	*Erwinia tasmaniensis*	GCF_000026185.1	80.82	722	1786	40.43
9	*Erwinia billingiae*	GCF_000196615.1	80.68	848	1786	47.48
10	*Erwinia typographi*	GCF_000773975.1	80.63	803	1786	44.96

**Table 3:** Antimicrobial susceptibility results of the isolate determined by Etest, disk diffusion, and Sensititre DKMGN panel.

Antimicrobial Agent	Etest MIC (μg/mL)	Disk Diffusion Zone (mm)	DKMGN MIC (μg/mL)	Interpretation
Ampicillin	—	10	—	R
Cefazolin	—	20	—	I
Cefotaxime	0.19	40	≤0.5	S
Ceftriaxone	0.25	28	—	S
Ceftazidime	0.125	30	≤0.5	S
Cefepime	—	40	—	S
Aztreonam	—	—	≤0.5	S
Imipenem	0.19	40	≤0.5	S
Meropenem	0.023	28	≤0.12	S
Ertapenem	0.008	42	≤0.12	S
Amoxicillin/clavulanic acid	—	—	≤4	S
Ampicillin/sulbactam	—	—	≤4/2	S
Piperacillin/tazobactam	2	30	≤1/4	S
Ceftazidime/avibactam	—	—	≤0.5	S
Ceftolozane/tazobactam	—	—	≤0.5	S
Amikacin	—	45	≤4	S
Gentamicin	0.25	40	≤0.5	S
Tobramycin	—	40	≤2	S
Ciprofloxacin	0.16	34	≤0.06	S
Levofloxacin	0.094	—	—	NA
Colistin	—	—	≤0.25	S
Tigecycline	—	—	≤0.25	NA
Trimethoprim/sulfamethoxazole	0.032	40	≤1	S

Abbreviations: MIC, minimum inhibitory concentration; S, susceptible; I, intermediate; R, resistant; —, not tested or not available; NA, not applicable.

## Data Availability

The whole-genome sequence of the isolate has been deposited in GenBank under accession number JAQISN000000000.1. The raw sequencing reads are available in the NCBI Sequence Read Archive under BioProject accession number PRJNA923122. All data supporting the findings of this study are available from the corresponding author upon reasonable request.

## Supplementary Materials

The following supporting information can be downloaded at: https://www.mdpi.com/article/10.3390/life15081227/s1, Table S1: KmerFinder results of the draft genome of the clinical *Erwinia* isolate; Table S2: Top 20 ANI matches against NCBI reference genomes from the *Erwiniaceae* family.
