# Supplementary material for: Bacteremia Caused by a Putative Novel Species in the Genus Erwinia: A Case Report and Genomic Analysis
Source: Life (Basel). 2025 Aug 3;15(8):1227. doi: 10.3390/life15081227 (PMC12387707; doi:10.3390/life15081227)
Supplement: Supplementary file 1 [file life-15-01227-s001.zip › life-3758833-supplementary.pdf]

Supplementary Table S1. KmerFinder results of the draft genome of the clinical *Erwinia* isolate

No	RefSeq accession	Species identified	Query coverage (%)	Template coverage (%)	Score	Description
1	GCF_024397315.1	*Erwinia persicina*	3.86	3.95	5710	*Erwinia persicina* strain SR15 chromosome, complete genome
2	GCF_000026185.1	*Erwinia tasmaniensis*	1.37	1.73	2027	*Erwinia tasmaniensis* strain ET1/99 complete chromosome
3	GCF_025490495.1	*Pantoea dispersa*	1.18	1.48	1751	*Pantoea dispersa* strain ML.8a3 chromosome, complete genome
4	GCF_022394795.1	*Pantoea* sp. Z09	0.82	1.23	1207	*Pantoea* sp. Z09 chromosome, complete genome

Supplementary Table S2. Top 20 ANI matches against NCBI reference genomes from the *Erwiniaceae* family

No	Species name	RefSeq accession	ANI (%)	Aligned fragments	Total fragments	Coverage (%)	Taxonomic status
1	*Pantoea coffeiphila*	GCF_016909495.1	90.28	1566	1786	87.68	Correct name
2	*Candidatus Erwinia dacicola*	GCF_001756855.1	82.77	328	1786	18.37	Pro-correct name
3	*Erwinia aphidicola*	GCF_037149315.1	82.75	994	1786	55.66	Correct name
4	*Erwinia rhapontici*	GCF_020683125.1	81.77	964	1786	53.98	Correct name
5	*Erwinia persicina*	GCF_019844095.1	81.60	959	1786	53.70	Correct name
6	*Erwinia sorbitola*	GCF_009738185.1	81.48	933	1786	52.24	Preferred name (not correct)
7	*Erwinia pyrifoliae*	GCF_002952315.1	81.06	691	1786	38.69	Correct name
8	*Erwinia amylovora*	GCF_043228865.1	80.93	688	1786	38.52	Correct name
9	*Erwinia piriflorinigrans*	GCF_001050515.1	80.86	686	1786	38.41	Correct name
10	*Erwinia tasmaniensis*	GCF_000026185.1	80.82	722	1786	40.43	Correct name
11	*Erwinia aeris*	GCF_041224955.1	80.82	791	1786	44.29	Preferred name (not correct)
12	*Erwinia plantamica*	GCF_043420595.1	80.81	787	1786	44.06	Preferred name (not correct)
13	*Erwinia billingiae*	GCF_000196615.1	80.68	848	1786	47.48	Correct name
14	*Erwinia typographi*	GCF_000773975.1	80.63	803	1786	44.96	Correct name
15	*Erwinia pyri*	GCF_030758455.1	80.53	789	1786	44.18	Preferred name (not correct)
16	*Erwinia phyllosphaerae*	GCF_019132875.1	80.41	730	1786	40.87	Correct name
17	*Erwinia toletana*	GCF_032164335.1	80.27	695	1786	38.91	Correct name
18	*Erwinia iniecta*	GCF_001267535.1	80.25	742	1786	41.55	Correct name
19	*Erwinia arboricola*	GCF_025527015.1	80.08	784	1786	43.90	Correct name
20	*Pantoea dispersa*	GCF_019890955.1	80.04	736	1786	41.21	Correct name

Extended list of the top 20 genomes showing the highest ANI values with the isolate, based on FastANI comparisons against all available genome assemblies from the family *Erwiniaceae*. This comparison includes species with synonyms, provisional names (e.g., *Candidatus*), and names not listed as correct in the List of Prokaryotic names with Standing

in Nomenclature (LPSN). While multiple *Erwinia* genomes appeared among the top hits, all exhibited ANI values below 83%, reinforcing the absence of a known species-level match. Taxonomic status was assigned according to the LPSN database (accessed July 2025). "Pro-correct name" refers to taxa proposed as correct names but not yet validly published under the ICNP.
